# The Controlled Release of Dexamethasone Sodium Phosphate from Bioactive Electrospun PCL/Gelatin Nanofiber Scaffold

**Published:** 2019

**Authors:** Fatemeh Rasti Boroojen, Shohreh Mashayekhan, Hojjat-Allah Abbaszadeh

**Affiliations:** a *Department of chemical and petroleum engineering, Sharif University of technology, , Tehran, Iran. *; b *Hearing Disorders Research Center, Loghman Hakim Hospital, Shahid Beheshti University of Medical Sciences, Tehran, Iran.*; c *Department of Biology and Anatomical Sciences, School of Medicine, Shahid Beheshti University of Medical sciences, Tehran, Iran.*

**Keywords:** Spinal cord injury, Dexamethasone, Electrospinning, Nanofiber scaffold, Controlled release

## Abstract

In this study, a system of dexamethasone sodium phosphate (DEXP)-loaded chitosan nanoparticles embedded in poly-ε-caprolacton (PCL) and gelatin electrospun nanofiber scaffold was introduced with potential therapeutic application for treatment of the nervous system. Besides anti-inflammatory properties, DEXP act through its glucocorticoid receptors, which are involved in the inhibition of astrocyte proliferation and microglial activation. Bovine serum albumin (BSA) was used to improve the encapsulation efficiency of DEXP within chitosan nanoparticles and to overcome its initial burst release. BSA incorporation within the chitosan nanoparticles increased the encapsulation efficiency of DEXP from 30% to 77%. The comparison between DEXP release profile from PCL/gelatin scaffold with and without chitosan nanoparticles revealed that the system of DEXP-BSA-loaded chitosan nanoparticles embedded in electrospun PCL nanofiber scaffold provided a more controlled release pattern of the loaded drug. The scaffolds properties in terms of structure, hydrophilicity, cell compatibility, mechanical property, and biodegradability were further investigated, which might show its potential application for the repair of spinal cord injury.

## Introduction

The ability of central nervous system (CNS) to regenerate is restricted due to the inhibitory environmental factors in the injury site as well as the inherent weakness in regeneration of CNS(1). In the site of injury, reactive astrocytes produce chondroitin sulfate proteoglycan, which is an inhibitory factor, while other glial cells create extracellular matrix (ECM), which results in the formation of glial scar as an inhibitory barrier to axonal regrowth (2). The use of different types of scaffold in an early phase of injury is mostly recommended for preventing the formation of glial scar (3). Biomaterial scaffolds can play a number of specific roles in nervous system regeneration. They can act as carriers delivering the therapeutic agents (e.g. dexamethasone (4), prednisolone (5), nerve growth factor (NGF) ((6), neurothrophin-3(NT-3) (7), brain-derived neurotrophic factor (BDNF) (8)), and stem cells (9).

The arranged structure of nanofiber scaffolds is more similar to the fibrous structures of native ECM in comparison with the isotropic structure of hydrogel. Electrospinning is one of the most fundamental methods to produce nanoscale fibers. Obviously, electrospun nanofiber scaffolds have sufficient porosity, high surface-to-volume ratio, and similar structural property to the native tissue (5).

Among all different types of synthetic polymers, PCL has been widely used for biomedical applications. Although PCL represents good mechanical properties, low hydrophilicity undermines cell attachment, proliferation, and migration (10). To deal with low biocompatibility of PCL, gelatin can be blended, which is a widely used protein in various fields of biomedical applications (11).

Dexamethasone (DEX), which is a glucocorticoid anti-inflammatory drug, suppresses inflammation responses in the nervous system, and affects astrocytes and glial cells through glucocorticoid receptors (12). This synthetic glucocorticoid binds to astrocyte glucocorticoid receptors, and inhibit astrocyte proliferation (12). In addition, it can lower the secretion of pro-inflammatory cytokines such as IL-1β, INF-γ, and TNF-α (12). In fact, glucocorticoids play an important role in a variety of intracellular activities such as metabolism and apoptosis. It is shown that glucocorticoids have various effects on different kinds of cells. For instance, glucocorticoids induce apoptosis in T lymphocytes, while they inhibit apoptosis in T cells, liver cells, and glioma cells (13). Apoptosis plays an important role in secondary spinal cord tissue damage. *In-vivo*, neurotrophin receptor p75 (p75^NTR^)-mediated oligodendrocyte apoptosis occurs after spinal cord injury (SCI). DEX reduces the expression of p75^NTR^, which results in the reduction of oligodendrocyte apoptosis and improvement in its function (14). Due to low growth and healing rate of the nervous tissue, the need of sustained release of DEX is vital. As a result of weak intermolecular interaction between DEX and PCL, the release rate of DEX incorporated in PCL nanofibers from PCL nanofibers is too fast (15). For controlled release of DEX, the nanoparticles carriers could be used to encapsulate drug and then were embedded in the nanofibers. However, dexamethasone sodium phosphate (DEXP), which is a sodium phosphate salt form of DEX has a low encapsulation efficiency due to its water soluble nature. Bovine serum albumin (BSA), which is the most abundant protein in the blood plasma that can bind to DEX by hydrogen binding and van der waals forces (16) can be applied to increase the encapsulation efficiency of the drug. 

In this study, DEXP-BSA-loaded nanoparticles were embedded in PCL nanofibers since the hybrid structure can act as physical barrier and therefore protect drug molecules against toxic solvents. Moreover, this system can eliminate the burst release of drug and cause a sustained release. 

**Figure 1:**
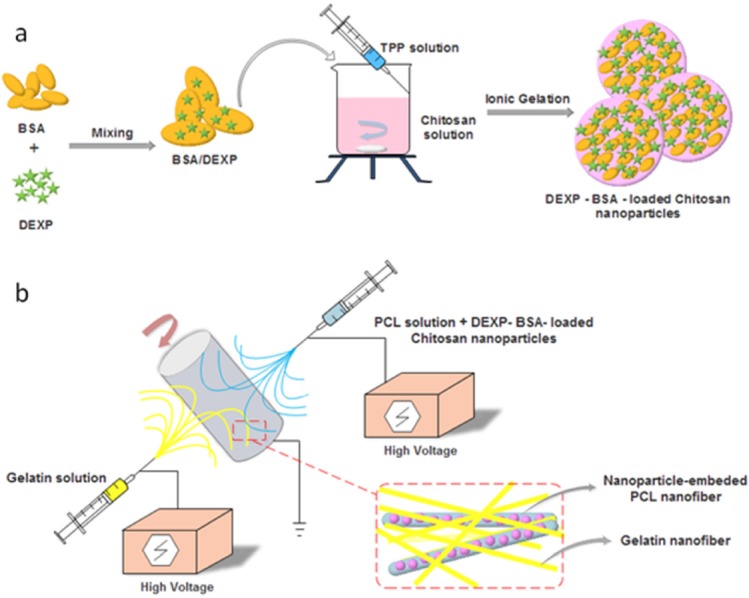
Schematic illustration of DEXP-BSA-loaded chitosan nanoparticles (NPs) (a) and electrospinning procedure for the fabricationof chitosan NPs-embedded PCL and gelatin nanofiber

The process of hybrid scaffold fabrication is shown in Figure 1. First, DEXP-BSA-loaded chitosan nanoparticles were fabricated and then suspended in PCL solution. Co-electrospining technique was used to fabricate hybrid scaffold. Gelatin solution from one side and DEXP-BSA-loaded chitosan nanoparticles-embedded PCL from another side were electrospun. To ascertain the value of the aforementioned hybrid nanofiber as the desired scaffold, the DEXP release behavior was monitored during time and the scaffold properties, such as tensile strength, hydrophilicity, biocompatibility and biodegradability were evaluated.

## Experimental

*Materials*

Poly-ɛ-caprolactan (PCL, M*w = *80*KDa*), gelatin (from porcine skin), dimethyl formamide (DMF), chloroform, MTT(3[4,5-dimethylthiazol-2-yl]-2,5-diphenyltetrazolium bromide), tripolyphosphate (TPP), chitosan (medium molecular weight), acetic acid, and glutaraldehyde andphosphate buffered saline (PBS) were all obtained from Sigma-Aldrich. Bovine serum albumin (BSA) was purchased from Merck. Dexamethasone sodium phosphate (DEXP) was obtained from Iran Hormone company. Trypsin, fetal bovine serum (FBS), Dulbecco’s modiﬁed Eagle’s medium/nutrient mixture F12 (DMEM/F12), and penicillin–streptomycin solution were obtained from GIPCO Invitrogen. 

*Dexamethasone-loaded chitosan nanoparticles preparation*

The ionic interactions between positive amine groups of chitosan and negative phosphate groups of TPP result in the formation of chitosan nanoparticles. The process of chitosan nanoparticle formation was based on our previous work with minor modification (17). Chitosan was dissolved in acetic acid (1% v/v ) to obtain 1.7 mg/ml chitosan solution. 

The pH of the chitosan solution was adjusted to 5.5. Dexamethasone sodium phosphate (DEXP) and BSA solution were mixed thoroughly with BSA/DEXP weight ratio of 4:5. Next, BSA-DEXP solution was added to chitosan solution with final BSA concentration of 0.2 mg/ml and final DEXP concentration of 0.25 mg/mL 12 mL of TPP solution was added to DEX-BSA-chitosan solution under 500 rpm stirring for 60 min. The nanoparticles were collected by 15000 rpm centrifuge at 4℃ for 60 min. Then, the nanoparticles were redispersed into DMF. Final solution was sonicated for 10 min to make a homogenous solution. 

*Electrospinning procedure*

1.2 gr of PCL was dissolved in 6 ml chloroform and 3ml of DMF under mild stirring for 2 h. 1mL of 3% (w/v) nanoparticle in DMF was added to PCL solution. 200 µL of tween-80 was also added to solution to create efficient binding between hydrophobic PCL and hydrophilic chitosan. To analyze the effect of chitosan nanoparticles on the DEXP release, the same amount of DEXP present in DEXP-BSA loaded chitosan nanoparticles was added to PCL solution (56 mg/mL DEXP) and electrospinning process was carried out. To prepare gelatin solution for electrospinning, 2.5 gr of gelatin was dissolved in 40% (v/v) acetic acid to get 25% (w/v) gelatin solution. 

To fabricate nanofiber scaffold,co-electrospinning technique was used with a Nano Model (Tehran, Iran) setup. 5mL syringe was filled with PCL or PCL solution containing DEXP-loaded chitosan nanoparticles and gelatin solutions. The flow rate of PCL and gelatin solutions was maintained at 0.5 mL/h. For gelatin solution, the applied voltage at the tip of syringe needle and the distance between needle and collector were 23 Kv and 15 cm, respectively. For PCL solution containing DEXP-loaded chitosan nanoparticles, the applied voltage at the tip of syringe needle and the distance between needle and collector were 16 kV and 17 cm, respectively. The speed of collector was fixed at 300 rpm.Air-dried composite nanofibers on the aluminum foil was placed in sealed desiccator including 10 mL aqueous glutaraldehyde solution with 25% (w/v) concentration for 24 h and crosslinking reaction took place in saturated glutaraldehyde vapor (18).

*Characterization*

*Particle size and size distribution of chitosan nanoparticles*

The suspension of nanoparticles was diluted to appropriate concentration with water, and test was done by dynamic light scattering (DLS method using a Zetasizer Nano S (Red badge) (Malvern Instruments, UK) to determine the size and size distribution of chitosan nanoparticles prepared by ionic gelation method. Triplicate samples were analyzed and the arithmetic mean value of the three was adopted.

*Drug loading and loading capacity analysis*

After preparation of DEXP-BSA loaded chitosan nanoparticles, nanoparticles were collected by 15000 rpm centrifuge at 4℃ for 60 min. After centrifugation, nanoparticles were settled from chitosan nanoparticle suspension, and the supernatant was collected. Drug concentration in supernatant was estimated by utilizing standard curve of DEXP concentration versus UV-absorbance at 242 nm. The UV-absorbance of DEXP was measured at λ_Max _*=* 242 nm by ultraviolet spectrophotometer. The drug encapsulation efficiency (LE) was calculated by equation 1. All measurements were done triplicate.

$\mathrm{EE\%}=\frac{M_{0}-M_{f}}{M_{0}}$

Equ. 1

Where *Mo* represents the total amount of drug existing in the solution before particle formation, and *M*_f_ is the amount of drug remaining in solution after ultracentrifugation in the form of free drug. In addition, DEXP loading capacity of chitosan nanoparticle was measured according to equation 2.

$LC\%=\frac{M_{0}-M_{f}}{M_{c}}$

Equ. 2

Where *Mo - M*_f_ is the amount of DEXP loaded in chitosan nanoparticles (µg), and *M*_c _ shows the amount of chitosan consumed to produce the chitosan nanoparticles.

*Scanning electron microscopy*

The mean size of electrospun nanofibers was analyzed by using a scanning electron microscope (SEM) utilizing AIS2100model (SERONTECHNOLOGIES Co.**, **South Korea) at 25 kV accelerated voltage. First, the samples were covered with gold for 90s by using SC7620model (QUORUMTECHNOLOGIES-EMITECH Co., UK).Nanofibers diameter was estimated from the SEM images with the aid of image J software.

*Drug release behavior from nanofibrous scaffolds*

Drug release from the scaffold was studied according to a common protocol described elsewhere (19). 100mg of dried scaffold with and without nanoparticles were immersed in 10ml phosphate buffer solution (PBS, pH = 7.4) in triplicate. The samples were transferred to sterile 6-well plates and put in a shaker incubator (80 rpm) at 37 ℃ for 3 weeks.At specified collection times, 4 mL PBS of each samples were taken from the dish and substituted with fresh PBS. The UV-absorbance of DEXP was measured at λ_Max _*=* 242 nm to estimate the DEXP concentration. The triplicate samples were analyzed. The total mass of released drug at time *i *was calculated from the following equation (equation 3):

$M_{i}=C_{i}V+\sum C_{i-1}V_{s}$

Equ. 3

Where *M*_i_ and *C*_i_ are total amount of released drug and released drug concentration in the solution at time i, respectively. *V*_s_ is the sample volume, and *V* is the total volume of release solution. Nanofiber scaffold without nanoparticles were considered as control sample.

*In-vitro degradation*

Electrospun samples were washed thoroughly with sterile distilled water and dried by vacuum drier. Each sample was cut into square shape and was precisely weighed. After weighing, the samples were put into sealed plates containing sufficient amount of PBS (pH = 7.4). The samples were settled in a shaker incubator at 37 ℃ (10). After 1, 2, and 3 weeks of incubation, electrospun scaffolds were taken out, washed with distilled water, and then put in a vacuum oven to be completely dried. The dried samples were weighed and degradation rate was calculated according to equation 4.

$\mathrm{degradation rate}=\frac{W_{0}-W_{f}}{W_{0}}\times 100$

Equ. 4

Where *Wo* is the initial weight of scaffold, and *W*_f_ is the weight of scaffold measured at every week interval of the scaffold degradation.

*Contact angle measurement*

The hydrophilicity of the scaffolds was investigated using OCA15 contact angle meter. 4 µL drop of DI water was put on the surface of the dried scaffold and the contact angle was measured. 

**Table 1:** Characterization of chitosan nanoparticles. (NPs)

**Sample**	**DEXP Conc. (mg/mL)**	**BSA Conc.(mg/mL)**	**EE (%)**	**LC (μg/mg)**	**Average NPs diameter (nm)**
DEXP-loaded NPs	0.25	0	33.4 ± 3.7	48.53 ± 11	78.8
DEXP-BSA-loaded NPs	0.25	0.2	77.2 ± 6.3	113.23 ± 14.5	149
P-Value	-	-	< 0.001	< 0.001	

**Table 2:** Mechanical properties of electrospun scaffolds after crosslinking process

**Sample**	**Elongation at break (mm/mm)**	**Young's Modulus (MPa)**	**Tensile strength (MPa)**
Gelatin	2.44±0.3	281±34	4.2±0.4
PCL	73.37±9	24.4±5	5.64±0.7
PCL/Gelatin	6.37±0.6	268.87±32	8.86±0.6

**Figure 2:**
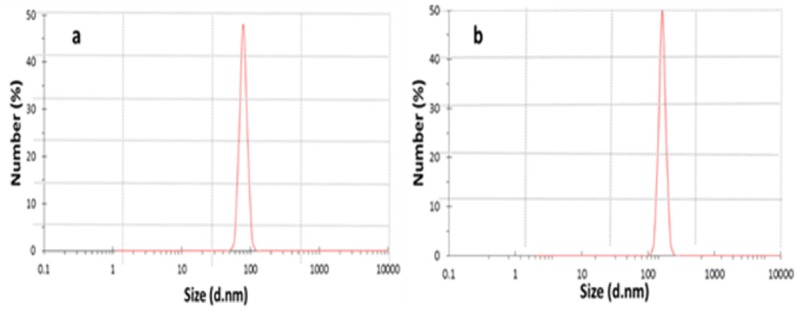
DLS analysis of chitosan nanoparticles (a) and DEXP-BSA-loaded nanoparticles (b)

**Figure 3:**
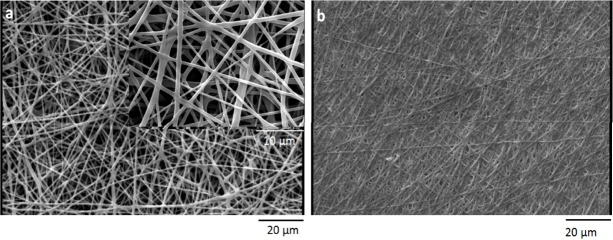
Scanning electron microscopy micrograph of PCL/gelatin a) before crosslinking b) after crosslinking

**Figure 4:**
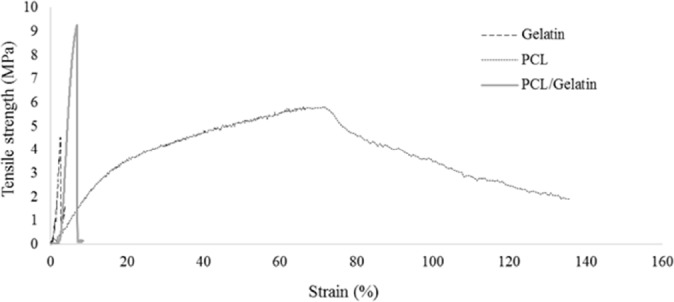
Mechanical properties of electrospun scaffolds after crosslinking process

**Figure 5:**
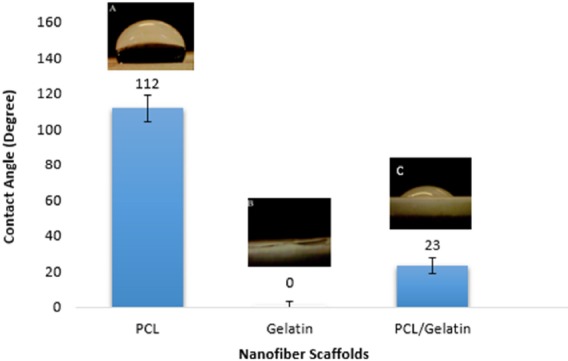
Contact angle measurement of nanofiber scaffolds

**Figure 6:**
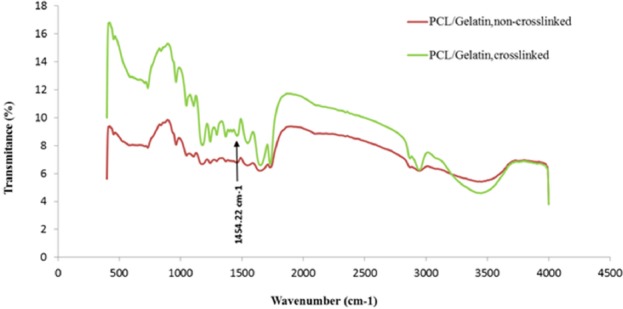
FTIR spectra of the scaffolds

**Figure 7:**
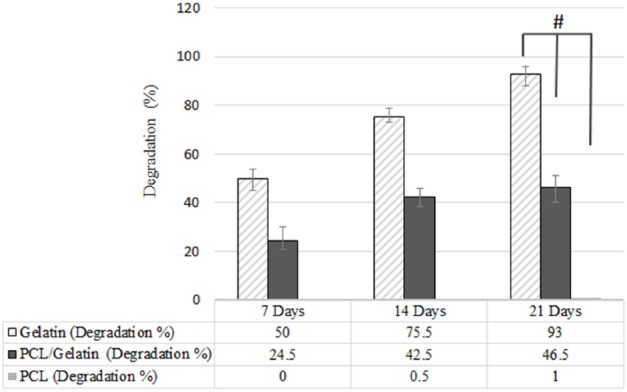
Biodegradation of PCL, PCL/gelatin and gelatin scaffolds, (*p ≤ 0.05)

**Figure 8:**
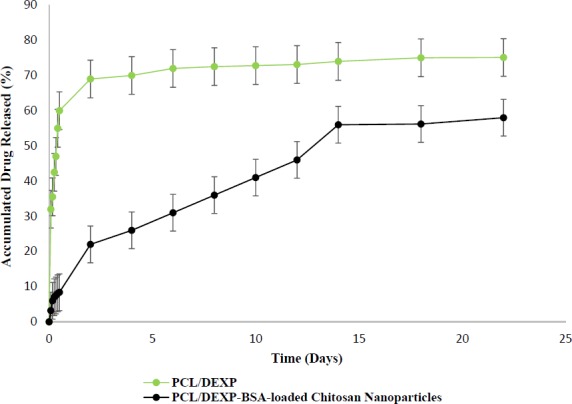
Release profile of DEXP from nanofiber scaffold with and without chitosan nanoparticles

**Figure 9:**
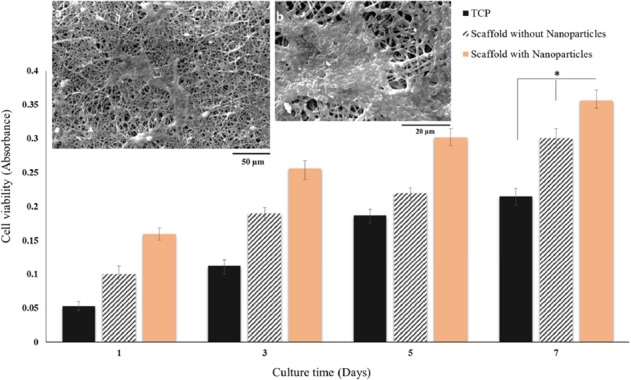
SEM images of BMSCs on PCL/gelatin nanofiber scaffolds containing DEXP-BSA-loaded chitosan nanoparticles at day 7 at different magnifications (a, b) and MTT viability assay of BMSCs cultured on PCL/gelatin nanofiber scaffolds with and without DEXP- BSA-loaded chitosan nanoparticles and TCP (control) at day 1, 3, 5 and 7 (c), (*p ≤ 0.05)

*FTIR analysis*

PCL/gelatin nanofibers were chemically analyzed before and after the crosslinking process. Functional groups of each sample and the chemical changes occurred during crosslinking reaction in gelatin structure were detected by Fourier transform infrared spectroscopy (FTIR) spectrophotometer using a spectrum RXI system in the range of 4000-400 cm^-1 ^at room temperature. Dried scaffolds were combined with KBr disk, and the uncrosslinked scaffold was considered as the control. 

*Mechanical property assay*

Nanofiber scaffolds were cut into rectangle (10×30 mm^2^). To characterize the mechanical behavior of the composite scaffold, a universal testing machine (Instron, model STM250, Iran) was applied at uniaxial stretching rate of 50 mm/min.

*In-vitro culture of mesenchymal stem cells*

Rat bone marrow-derived mesenchymal stem cells (BMSCs) were obtained from female rats (200-250 g: Shahid Beheshti medical school, Tehran, Iran). The procedure was approved by the Ethical Committee of Shahid Beheshti University Medical School (Tehran, Iran). The bone marrow was removed from the femurs and the tibias of sacrificed rats. Basically, BMSCs were isolated according to their ability to adhere to tissue culture plate. Briefly, the whole bone marrow were cultured on tissue culture flask in DMEM/F12 containing penicillin, streptomycin, L–glutamine, and 10% FBS at 5% CO_2_, 95% air humidity and 37 ℃ . The medium was changed on day 1, and the non-adherent cells were discarded. After day 1, the medium was replaced daily until they reached 70% cell confluency. The BMSCs at passage four were used for MTT assay (20).

*Analysis of cell morphology, viability and proliferation*

To analysis the scaffold biocompatibility, MTT assay was employed. Rat BMSCs (5000 cells/cm^2^) were cultured on 96- well tissue culture plate (as control) as well as nanofiber PCL/gelatin scaffold with and without DEX-loaded chitosan nanoparticles. The viability of BMSCs seeded on nanofiber scaffold was indicated by MTT assay after 1, 3, 5, and 7 days. After each interval, the cells were washed by sterile PBS. 100 µL of fresh DMEM/F12 medium and 10 µL of 5 mg/mL MTT were added to each well. The plate was incubated for 2-4 h at 37 ℃ . After the appearance of purple precipitate, medium was discarded, followed by adding 100 µL of DMSO, as a detergent reagent. The plate was covered and left in the dark at ambient temperature for 2 h. The content of each well was transferred to new 96-multiwell plate, and its absorbance was measured at 570 nm utilizing Biotek ELISA reader. The morphology of MSCs seeded on the nanofiber scaffold was monitored by SEM (AIS2100 model (Seron Technologies Co.**, **South Korea)). Briefly, BMSCs cultured on the scaffold were fixed by 2.5% glutaraldehyde for 2-4 h followed by dehydration using gradient ethanol concentration (60, 70, 80, 90, 100%) for 75 min. Eventually, the prepared scaffolds were covered with thin gold layer and observed by SEM.

*Statistical analysis*

All the experiments and analysis were conducted in triplicate, and the results were reported as arithmetic mean ± standard deviation (SD). Statistical analysis was performed using SPSS.18 statistical software. *p* < 0.05 were considered statistically significant. 

## Results and Discussion

*Physical characterization of nanoparticles and nanofibers*

By using ionic gelation method, DEXP-loaded and DEXP-BSA-loaded chitosan nanoparticles were synthesized. Different parameters such as the molecular weight of chitosan, TPP and chitosan concentration, the pH of chitosan solution and the rate of stirrer affect not only the particle size but also the drug loading efficiency (21, 22). The combination of optimum parameters results in higher drug loading and suitable size of chitosan nanoparticles, which leads to electrospinning nanofibers with more homogenous distribution of nanoparticles. In recent work done by Vakilian *et al*., the optimum condition was employed to synthesize chitosan nanoparticles with a narrow particle size distribution (17). In the present research, the same condition was applied, and the obtained results by DLS exhibited a mean diameter of 149 nm and 78.8 nm for the nanoparticles with and without drug loading, respectively (Figure 2).The loading capacity and encapsulation efficiency of DEXP within the chitosan nanoparticles without using BSA was measured to be 48.53±11 and 33 ± 3.7, respectively. BSA was added to DEXP solution prior to nanoparticles fabrication to enhance the encapsulation efficiency of the drug. Albumin, which is the most abundant plasma protein (23), has different binding sites. The most important binding sites of albumin are denoted as site I, site II, and site III (16). DEXP primarily binds to BSA at site III. Van der Waals forces and hydrogen bonding are involved in the interaction between DEXP and albumin (16). After BSA incorporation, the drug loading capacity and encapsulation efficiency was increased to 113.23±14.5 µg DEXP per mg of chitosan and 77.2±6.3%, respectively (Table 1). BSA acts as a bridge between chitosan chain and DEXP molecules. The interaction between BSA and chitosan is electrostatic, and the interaction between DEXP and BSA is hydrogenic or van der Waals. These kinds of interaction play an important role in increase of the drug encapsulation efficiency.

Before crosslinking process, the fiber diameter of nanfibers was characterized by using SEM micrographs of electrospun scaffold. The average size of PCL nanofibers was 793±20, and the average size of gelatin nanofibers was 263±10. To increase the stability of gelatin nanofibers in aqueous medium, the electrospun scaffolds were crosslinked by using saturated glutaraldehyde vapor. After crosslinking process, the gelatin nanofibers blended together at contacting points (Figure 3).

*The mechanical properties of electrospunscaffold*

The mechanical properties of PCL, gelatin and those of hybrid scaffold are shown in Figure 4. Crosslinking process creates a gelatin substrate in which PCL nanofibers embedded. The mechanical properties of electrospun scaffolds were analyzed according to the stress-strain curve. The corresponded Young›s modulus, tensile strength, and elongation at break are summarized in Table 2 Co-electrospinning of PCL and gelatin increased the Young›s modulus of the hybrid nanofibers compared to the pure PCL nanofibers. Elongation at break of composite nanofibers was higher than gelatin nanofibers due to the presence of PCL nanofibers and their elastic behavior. In terms of tensile strength, PCL/gelatin scaffold showed higher strength than pure PCL and gelatin. 

*The hydrophilicity analysis of the scaffolds*

One of the important parameters that should be characterized is the hydrophilicity of the scaffold. While hydrophobic polymers can trigger immune responses due to the adhesion of monocyte at the surface of polymer, hydrophilic polymers decrease monocyte interaction and the formation of foreign body giant cell (24). Moreover, it is desired to increase the surface hydrophilicity of polyesters in order to improve the cell-material interaction. As shown in Figure 5, co-electrospining of PCL and gelatin decreases the contact angle of the scaffold from 112˚̊to 23˚ . 

*FTIR analysis*

To characterize the functional groups of PCL and gelatin and to study the chemical interaction occurred between glutaraldehyde and gelatin, FTIR spectra of the scaffolds were obtained. As shows in Figure 6, several characteristic infrared (IR) bands of PCL were observed at 2949 cm^-1^ and 2865 cm^-1^ (asymmetric and symmetric –CH2 stretching), 1726 cm^-1^ (C = O strertching), 1293 cm^-1^ (C–O and C–C stretching), 1240 cm^-1^(asymmetric C–O–C stretching), and 1170 cm^-1^ (symmetric C–O–C stretching) (25). Similarly, the IR spectrum of gelatin represented important bands related to the amino groups of gelatin including 1636-1640 cm^-1^ (C = O stretching) associated to amide-I , 1542-1544 cm^-1^(N-H bending and C-H starching) related to amid-II, 1240 cm^-1^ (C-N stretching and N-H phase bending) associated to amid-III, and 3300 cm^-1^ (N-H starching vibration) associated to amide A (26). Chemical reaction of aldehyde groups of glutaraldehyde with amino groups of gelatin occurs, and the formation of aldimine linkage is the result of this reaction (27).The presence of the aforementioned linkage is justified by a peak at 1450 cm^-1 ^(28). Changing the color of samples happened during the crosslinking procedure is due to the appearance of aldimine linkage (26). As shown in Figure 6, FTIR spectra of the crosslinked scaffold illustrate a small peak at 1450 cm^-1^ in comparison to un-crosslinked scaffold.

*Biodegradability result*s 

One of the most crucial factors to design scaffold is biodegradability. Degradation rate of scaffold should be controlled in adequate manner in order to allow tissue regeneration and remodeling (29). In fact, there should exist a balance between tissue regeneration and scaffold degradation rate. Although PCL represents good mechanical properties, it has too slow degradation rate to be suitable for neural tissue engineering. Gradual degradation results in low nerve regeneration due to barrier structure of the scaffold (30).

While PCL degradation mechanism occurs by hydrolyzing PCL ester groups (31), gelatin degradation occurs via both the hydrolysis and enzymatic reaction. In addition to fast degradation rate of gelatin in physiological condition, the sensitivity of gelatin to water and its water solubility is another obstacle to the use of gelatin in tissue engineering application (32). To solve this problem and control the degradation rate, several strategies have been introduced (33-35). One of these solutions which was applied in this research is the use of glutaraldehyde as crosslinker (33). The weight loss of scaffolds was examined for 3 weeks. As shown in Figure 7, the addition of gelatin increased the degradation rate of PCL scaffold due to high biodegradability of gelatin. These results are in accordance with those data reported by Ghasemi *et al*. (10). 

*DEXP release behavior from nanofibrous scaffolds*

Recently the application of scaffold so as to deliver proteins, growth factors, and other therapeutic agents to the SCI site has attracted much attentions (36). However, there exist drug restriction to pass the blood spinal cord barrier from blood circulation (37). The experiments have shown that large molecules with molecular weight grater then 500 Da are totally rejected and few molecules with molecular weight less than 500 Da can pass the blood spinal cord barrier. Therefore, it is beneficial to use scaffold as drug carrier or growth factor carrier to avoid the rapid disappearance of drugs or growth factors in the site of injury and deliver the desired drug and growth factor to SCI site (39). As discussed elsewhere, scaffolds can act as carrier to accelerate nerve regeneration or to prevent the secondary injury response in SCI (40). Utilization of DEXP has been already investigated to prevent the migration and activation of astrocyte cells (12). Moreover, the release of DEXP, as an anti-inflammatory drug, can modulate the body inflammation response for long-term application of biomaterials (41). Overall, DEXP may consider to play multiple roles in SCI. As previously mentioned, it prevents the apoptosis of oligodendrocytes (14) as well as the activation of astrocyte cells (12). Moreover, it affects the immune system and suppresses the inflammation response by reducing numerous pro-inflammatory cytokines (38,41).

The release profile of DEXP from PCL/gelatin scaffolds is shown in Figure 8. There is a significantly obvious difference between the drug release profiles of scaffolds with and without chitosan nanoparticles. The direct incorporation of DEXP into the PCL solution resulted in an initial burst release due to the hydrophilicity and hydrophobicity nature of DEXP and PCL, respectively, which cause phase separation of PCL-DEXP. The hydrophobic nature of PCL caused the migration of DEXP from bulk to the surface of PCL nanofibers, which leaded to the reduction in the distance between DEXP and release medium. The short path between DEXP and release medium contributed to fast release rate of drug. The presence of chitosan nanoparticles in nanofibers increased drug affinity to remain in the scaffolds due to the hydrophilicity of chitosan (42). Additionally, hydrogen and van der Waals forces between DEXP and BSA molecules in the chitosan nanoparticles did not allow DEXP to leave the nanoparticles quickly. Hence, chitosan nanoparticles and PCL nanofibers are considered to be two barriers against the DEXP release. DEXP release profile from chitosan nanoparticles-incorporated PCL nanofibers (PCL/DEXP-loaded chitosan nanoparticles) exhibited a slow and sustainable release in comparison to the nanofibers without chitosan nanoparticles (PCL/DEXP). 

As shown in Figure 8, the percent of accumulated drug released from nanofiber scaffold containing nanoparticles reached 60% after day 14, and the drug release rate after this day was too slow to be considered. There exist different factors controlling the drug release such as compatibility of drug and matrix, drug molecular weight, and scaffold structure. One of the important factors is polymer crystallinity that directly affects the drug release profile (43). PCL is a semi crystalline polymer with both amorphous and crystalline region (44). The limited penetration of water in PCL structure and the entrapment of drug result from crystalline regions of PCL. Due to low degradation rate of PCL the release mechanism of DEXP from scaffold were controlled by chitosan nanoparticle degradation rather than PCL degradation (17).

*In-vitro biocompatibility of nanofiber scaffold*

A SEM image of BMSCs on PCL/gelatin with DEXP-loaded chitosan nanoparticles at day 7 is shown in Figure 9 (a, b). The scaffolds proved to support the attachment, spread, and growth of BMSCs.

MTT assay was accomplished so as to evaluate the *in-vitro* biocompatibility of the nanofiber scaffolds using BMSCs. The cells were cultured on tissue culture plate (TCP) as control. To evaluate the effect of DEXP released from nanofiber on the cells, BMSCs were seeded on scaffold containing DEXP-loaded chitosan nanoparticles as well as scaffolds containing chitosan nanoparticles without DEXP.As shown in Figure 9 (c), by comparing viability of the cells cultured on scaffolds with and without DEXP, it can be revealed that DEXP has increased the cell proliferation. These results are in accordance with those reported by Medrao *et al*. showing that BMSCs cultured on chitosan-gelatin hydrogel in the presence of DEXP showed higher viability and proliferation compared with the ones seeded on chitosan-gelatin hydrogel without DEXP. However, the mechanism of this process is not recognized very well (45). In another study, Sun *et al.* fabricated DEX-loaded graphene oxide chitosan nanocomposite and showed that the cytotoxicity of DEX was highly dependent on both the drug concentration and release profile (46). According to the reported MTT results in this study, the concentration and the profile of DEXP released from the scaffold did not exhibit any cytotoxicity on BMSCs.

## Conclusion

In conclusion, co-electrospining of PCL containing DEXP-BSA-loaded chitosan nanoparticles and gelatin result in potentially suitable scaffold with desired mechanical properties, hydrophilicity and controlled release of DEXP, which might be used as a bridging biomaterial construct that allows the axons to grow through besides avoiding glial scar formation by inhibiting astrocyte proliferation and reducing the oligodendrocyte apoptosis for the repair of SCI. 

The result of MTT assay and SEM images support the biocompatibility of the hybrid scaffold. This scaffold also provides a delivery system of sustained release of the drugs and growth factors to be used in tissue engineering and regenerative medicine. Our ongoing studies are focused on delivering differentiation factors to neural stem cells cultured on the scaffolds in order to differentiate them into oligodendrocyte cells. 
